# Dr. Kinglake, in Reply to Mr. Pope, on the Gout

**Published:** 1807-09

**Authors:** Robert Kinglake

**Affiliations:** Taunton


### *To the Editors of the Medical and Physical Journal.*

GENTLEMEN,

YOUR correspondent Mr. Pope, in the last Number of your Journal, makes a profession of being solely actuated by a motive of benevolence in calling the public attention to what he considers as evidence of the "bad effects of the cooling treatment of gout." So confident does Mr. P. appear to be of the great importance of his communication, that he seems to anticipate no small share of influence from his narrative of the alleged injury "in inducing me to renounce a practice often fraught with the most mischievous and dreadful consequences."

That Mr. P. really believes what he asserts does not become me to question, but that the speculations he founds on the statement of facts he has given, are entitled to admission, is a point of considerable doubt. Mr. P. enters on his subject with a declaration of "having had three instances of the bad effects of the cooling treatment of gout," but for want of having taken notes of two of them, he has not felt competent to detailing them to the public. The case stated, therefore, was daily *noted*, and consequently assumes the authenticity of a medical report. This *notable* case then, it should seem, originated from a "trifling fling sprain of the ankle," which had occurred "three days before an agonising pain of the stomach, attended with nausea, vertigo, and a pulse not exceeding 54 strokes in a minute" presented. The local effects of the sprain at length proceeded to "violent pain, and considerable inflammation," yet no abatement is said to have happened to the constitutional symptoms. On the sixth day, however, "rags wetted in cold water, mixed with a small quantity of Goulard's extract, were applied to the *(sprained)* ankle and foot," which afforded almost immediate relief: but it is observed, the relief was "short lived," the aforementioned symptoms recurring in the evening. Our author, however, does not trouble himself with any speculation on the reason why the "short lived" relief was not protracted to a longer period. It is, indeed, not very likely that an *effect* will long survive its *cause*. The cooling application is allowed to have procured the relief that was experienced, but its employ would appear to have been insufficiently continued, which probably led to the "short lived" relief afforded by it.

The impartial reader must perceive an unaccountable strangeness in Mr. P. not resorting to the remedy that had proved advantageous; on the contrary, in his preferring to excite by warm water and other internal stimulants, parts but recently, and for a short time only, relieved from the inflammatory effects of a sprain. Mr. P. closes the detail of his *notable* case by observing, what should have been the first object of notice, that the lady who was the subject of it, had "paternally inherited the gout," and had been periodically attacked with the disease during twenty years. This circumstance warranted our author in concluding, that what by his own confession was primarily an affair of *sprain*, eventually became gout. This persuasion was sufficient in his estimation, (whether "reasonably" or not, as he assumes, is not quite clear,) to authorise an assiduous endeavour to drive the gouty enemy from the system, by a stimulating mode of treatment. Mr. P. indeed asserts, that the difficulties of his patient were not to be surmounted, until what he terms the "morbific action" had run its career: whether this said action was on the extremities, the stomach, or the brain, is not stated.

These, if I mistake not, are the leading merits of a case which formed *one* out of *three* deserving to be *noticed*, or rather, of which *notes* were actually taken. If the case thus honoured by public *notice*, be superior in its claim to attention attention to the *other two*, nothing surely will have been lost in not *noticing* them; whilst injury might be done to the reputation of a treatment intended to be opposed, by the *insidious* intimation given of their existence.

In return, I can truly aver that I have no personal "disrespect" to Mr. P.; but it behoves me likewise to affirm, that it is not in my power to feel the smallest *respect* for the kind of case with which he has thought proper to censure the cooling treatment of gout, nor for the apparently hopeless prejudice that renders him so *unthinkingly* averse to the practice. I am open to conviction on all questionable subjects, but am, and ever must remain, inaccessible by any such authority as Mr. P's case furnishes.

I am not disposed to trespass on your book by a lengthened explanation of the support which my opinion of the nature and appropriate treatment of gouty inflammation, derives from the real merits of Mr. P's case. It will be sufficient for that purpose to observe, that sprained ligaments and tendons, often induce distressing constitutional symptoms; amongst others, *locked jaw*, and even general convulsions, are not very rare occurrences. "Agonising pain at the stomach, nausea, and vertigo;" these may well arise from such a cause, and that they did originate from it in the case under consideration, is evinced by the fact that those symptoms *followed*, and did not *precede* the infliction of local violence.

In the course of a few weeks, I shall submit to the public in a work now in considerable forwardness in the press, a large *additional* stock of facts on the cooling treatment of gout, in the number of which, it is possible there may be arguments sufficiently cogent in favour of the practice, to overcome Mr. P's prejudice against it, and to convince him, (if open to conviction) that crude and common place notions on a medical subject, are "fraught with more mischievous and dreadful consequences" than are justly imputable to the cooling treatment of gout.

I am, &c.

ROBERT KINGLAKE.

*Taunton, July* 9, 1807.

